# Social noise interferes with learning in a volatile environment

**DOI:** 10.1038/s41598-019-44101-w

**Published:** 2019-05-20

**Authors:** Dong Woo Shin, Jaejoong Kim, Bumseok Jeong, Ko Woon Kim, Geumsook Shim, Taekeun Yoon

**Affiliations:** 1 Graduate School of Medical Science and Engineering, Korea Advanced Institute of Science and Technology (KAIST), Daejeon, Republic of Korea; 2 KAIST Institute for Health Science and Technology, KAIST, Daejeon, Republic of Korea; 3 Department of Neurology, Chonbuk National University School of Medicine, Jeonju, Republic of Korea; 4 KAIST Clinic Pappalardo Center, KAIST, Daejeon, Republic of Korea

**Keywords:** Computational models, Decision, Learning algorithms, Emotion, Social neuroscience

## Abstract

To learn through feedback, feedback should be reliable. However, if feedback is blurred by irrelevant social information, learning in a volatile environment, which requires fast learning and adaptation, might be disturbed. In this study, we investigated how feedback with social noise interferes with learning in a volatile environment by designing a probabilistic associative learning task in which the association probability changes dynamically, and the outcome was randomly blurred by an emotional face with incongruent valence. Learning in this situation was modelled by HGF-S such that emotionally incongruent feedback induces perceptual uncertainty called social noise. The Bayesian model comparison showed that the HGF-S model explains the subjects’ behaviour well, and the simulation showed that social noise interrupts both learning the association probability and the volatility. Furthermore, the learning interruption influenced the subsequent decision. Finally, we found that the individual difference in how the same emotionally incongruent feedback induces social noise in varying degrees was related to the differences in event-related desynchronization induced by happy and sad faces in the right anterior insula, which encodes the degree of emotional feeling. These results advance our understanding of how feedback with emotional interference affects learning.

## Introduction

Feedback is essential for learning whether our current belief or action is correct or incorrect. Ideally, if feedback clearly informs whether our choice or belief is correct or incorrect, we can learn from such feedback and change our belief or decision based on this information. However, if feedback conveys an ambiguous message, we are uncertain whether we should change our belief or behaviour. In daily life, we receive uncertain feedback when irrelevant social information distracts from the correct feedback information. For example, we ask a shopkeeper at a fruit store whether the new or original strawberries are more delicious, and the shopkeeper reports that the new strawberries are more delicious than the original strawberries but displays a negative facial expression. While the shopkeeper informed us that the new strawberries are more delicious, we cannot be certain due to the inconsistency between the valence of his words and his facial expression.

This type of distraction has been studied from the perspective of emotional interference using emotional Stroop or affective go/no-go tasks^1^, and emotional interference has been shown to increase error or the response time of decision-making. While these types of studies investigated whether emotional interference has an effect before decision-making, how emotional interference occurring while receiving feedback influences learning is unknown. Therefore, we investigated this question using feedback with distracting (emotional interference) or consistent social information. In particular, we were interested in investigating a dynamically changing environment in which individuals should learn quickly from feedback to adapt their belief or behaviour in response to the changing environment. In this environment, learning involves not only how the state of the environment changed but also how ‘volatile’ the environment is^2,3^. We expected that feedback with emotional interference would deteriorate learning about the changing environmental status and learning about the volatility of the environment.

**Figure 1:**
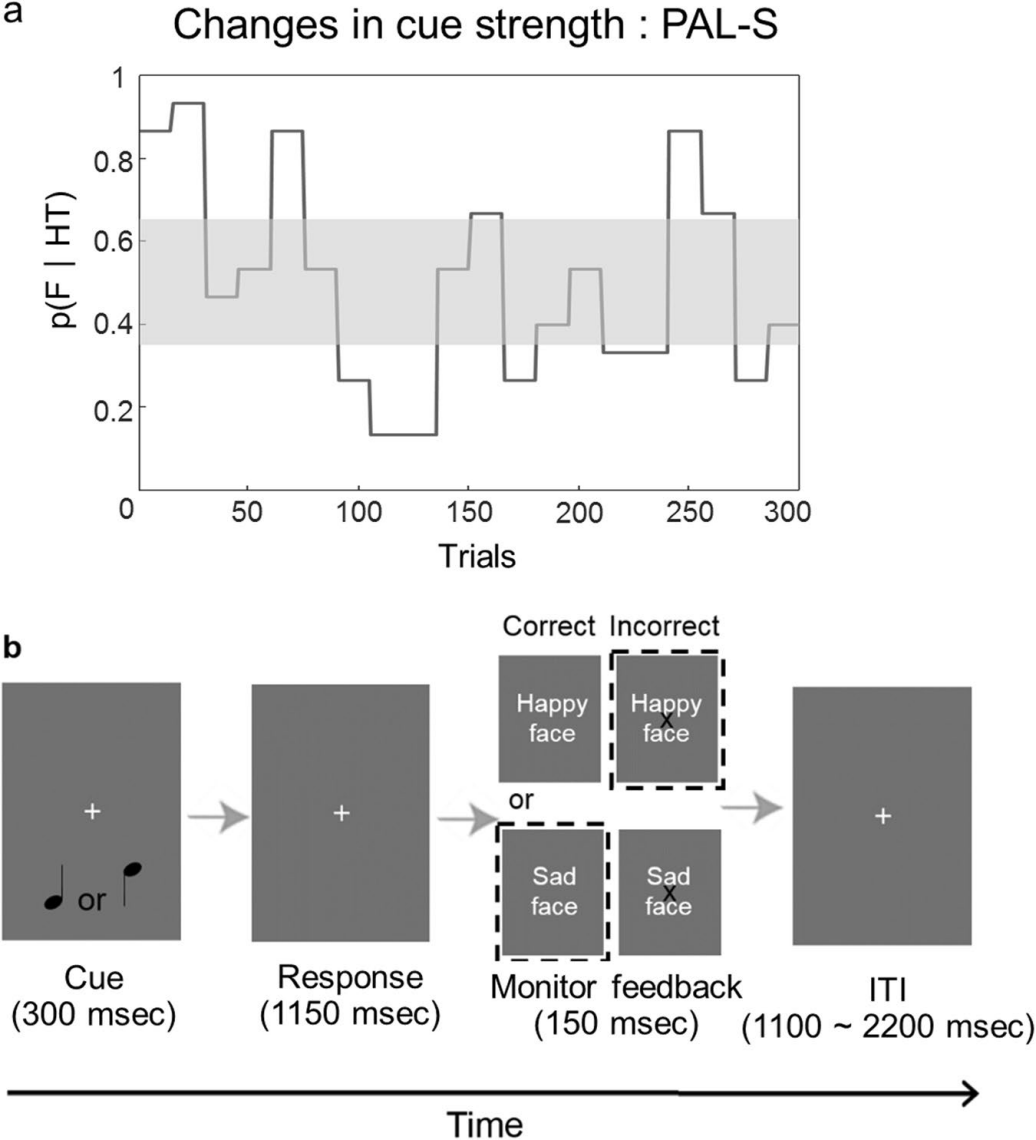
PAL-S task structure. (**a**) The probability of a female (F) appearing after a high tone (HT) in PAL-S is defined as p (F|HT), and the change in p (F|HT) over time is shown. The grey box represents the range of high irreducible uncertainty. (**b**) The experiment consisted of an experimental procedure comprising fixation and auditory cues, response, feedback confirmation, and a variable inter-trial interval. The subjects were instructed to judge the correct answer by whether an “X” mark was presented on the images. The emotional faces used as feedback were randomly displayed. The feedback is classified into congruent or incongruent pairs according to the relationship between the correctness of the answer and the emotional face shown. The dashed rectangles represent incongruent pairs but did not appear in the experiment. ITI: inter-trial interval.

In this study, we designed a task in which the agent learns a probabilistic relationship between a cue and an outcome while the relationship continuously changes during the task, rendering the probabilistic environment volatile. Importantly, feedback with emotional interference was randomly presented, and we named this task probabilistic associative learning with social noise (PAL-S; see Fig. 1 and Methods). To emotionally interfere with the feedback, emotional faces were presented along with the feedback such that the valences of the emotional faces were either congruent (e.g., correct response with a sad face) or incongruent (e.g., incorrect response with a happy face) with the outcome. Notably, we named these types of feedback emotionally incongruent feedback and emotionally congruent feedback. We hypothesized that incongruency between the valence of the emotional face and the valence of the feedback could induce perceptual uncertainty regarding the feedback, which we named social noise, and that this social noise could decrease the learning of the dynamically changing association probability between the cue and outcome. This hypothesis was tested as follows. First, before the computational modelling of the behaviour, we expected that the influence of social noise on the subjects’ learning could be reflected in the subjects’ behaviour patterns. In particular, we hypothesized that social noise would decrease learning by feedback and that this decrement in learning would influence the decision change in the subsequent trial.

**Figure 2:**
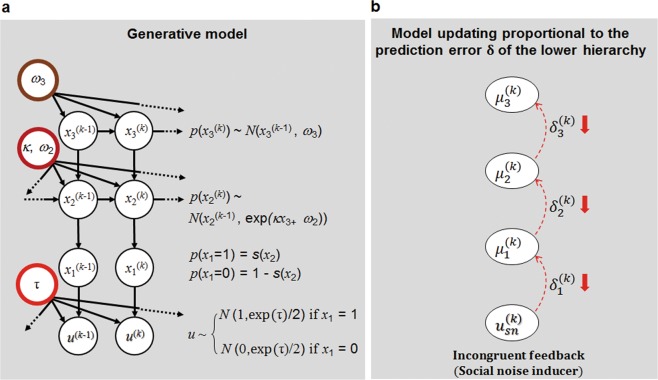
(**a**) Schematic representation of the three-level hierarchical Gaussian filter with social noise (HGF-S) model^8,23^. The HGF-S model assumes that an agent has an internal model of the environment that generates sensory input at trial *k* (*u*^(*k*)^) structured hierarchically as ${x}_{1}^{(k)}$, ${x}_{2}^{(k)}$, ${x}_{3}^{(k)}$. At the lowest level, i.e., *x*_1_, due to perceptual uncertainty (social noise induced by incongruent feedback), *u*^(*k*)^ is generated probabilistically following a Gaussian distribution centred at the actual environmental states of the lowest level (*x*_1_) with variance exp(*τ*)/2. *ω*_3_: tonic volatility at level 3 (brown circle), *ω*_2_: tonic volatility at level 2 (dark red circle), *κ:* phasic component at level 2 (dark red circle), *τ*: perceptual uncertainty (light red circle), *k*: time point, *u*^(*k*)^: sensory input at time point *k* (because *u*^(*k*)^ is the observed parameter, *u*^(*k*)^ is shaded). (**b**) Effect of social noise on model updating. In the HGF model, the updating of the generative model at each level is proportional to the prediction error occurring at the level below in the hierarchy and is weighed by the relative precision between the prediction at the higher level and the precision of the bottom-up prediction error. In the HGF-S model, incongruent feedback (${u}_{sn}^{(k)}$ in this figure) induces social noise, which decreases the model updating $({\delta }_{1}^{(k)}$, prediction error) towards the input represented by the difference between the posterior mean (${\mu }_{1}^{(k)}$) and the prediction (${\hat{\mu }}_{1}^{(k)})$ at level 1. Because the model updating at the first level is decreased, the model updating at level 2, which is proportional to the model updating at level 1 (${\delta }_{1}^{(k)})$, is also decreased; similarly, the model updating at level 3 is decreased. Therefore, social noise decreases model updating throughout the hierarchy.

Second, we explicitly modelled the subjects’ learning in a volatile environment using the Hierarchical Gaussian Filter model, which is known to explain learning behaviour in a volatile environment by assuming that an agent has an internal generative model of the environment with a multiple hierarchy that causes sensory input. In this model, the internal generative model encodes beliefs about environmental states (i.e., the association probability in our task) and beliefs about the volatility of the environment (i.e., how fast the association probability changes in our task). Furthermore, the influence of social noise on learning was modelled by introducing social noise as perceptual uncertainty induced by emotionally incongruent feedback in the HGF model, which was named HGF with social noise (HGF-S). In particular, the perceptual uncertainty parameter *τ* in this model represented the degree to which the incongruent emotional faces acted as social noise (see Fig. 2).

Third, we hypothesized that the individual differences in the interference by the emotional faces could be related to the degree to which neural responses were evoked by the emotional faces. Therefore, we assumed that an individual subject’s sensitivity to emotional faces could be represented by differences in event-related desynchronization (ERD) in the contrast between happy and sad faces (hereafter, we refer to this variable as ‘ERD difference’). This ‘ERD difference’ represents the amount of brain resources used to process positive versus negative emotional faces. To quantify the ‘ERD difference’ in the time-frequency domain in each subject’s brain under conditions separate from PAL-S, we performed magnetoencephalography (MEG) recordings during an emotional face perception task independent of PAL-S.

## Results

### Effect of social noise on decision change in the subsequent trial

We hypothesized that if the emotionally incongruent feedback induces social noise, the generative model updating induced by this feedback would be smaller than the model updating induced by emotionally congruent feedback, which would affect the subjects’ decision-making in the subsequent trial. More specifically, we hypothesized that this effect would differ depending on whether the subjects made a correct or incorrect choice. If the subjects made an incorrect choice and emotionally congruent feedback (a sad face) was given, the belief of the subjects would be biased against the previous choice, and thus, the subjects would likely change their decision. However, if emotionally incongruent feedback (a happy face) was given following an incorrect choice, such social noise would interfere with belief updating and might result in less decision change. In contrast, a correct choice would strengthen the belief regarding the previous choice, which might result in a choice consistent with that made in the previous trial. However, emotionally incongruent feedback would also interfere with this belief updating, and hence, the subjects would be less likely to make a choice consistent with that in the previous trial. These influences of social noise on decision changes, which we named the SN_d effect (effect of Social Noise on Decision change), were quantified for each subject as an interaction effect between emotionally congruent feedback and the correctness of the choice on the decision change ratio, which was formulated as follows:

$$SN\_d=(DC\_WC-DC\_WI)-(DC\_CC-DC\_CI)$$

*DC*_*WC*: Decision Change ratio after Wrong choice with emotionally Congruent feedback. *DC*_*WI*: Decision Change ratio after Wrong choice with emotionally Incongruent feedback. *DC*_*CC*: Decision Change ratio after Correct choice with emotionally Congruent feedback. *DC*_*CI*: Decision Change ratio after Correct choice with emotionally Incongruent feedback.

**Figure 3:**
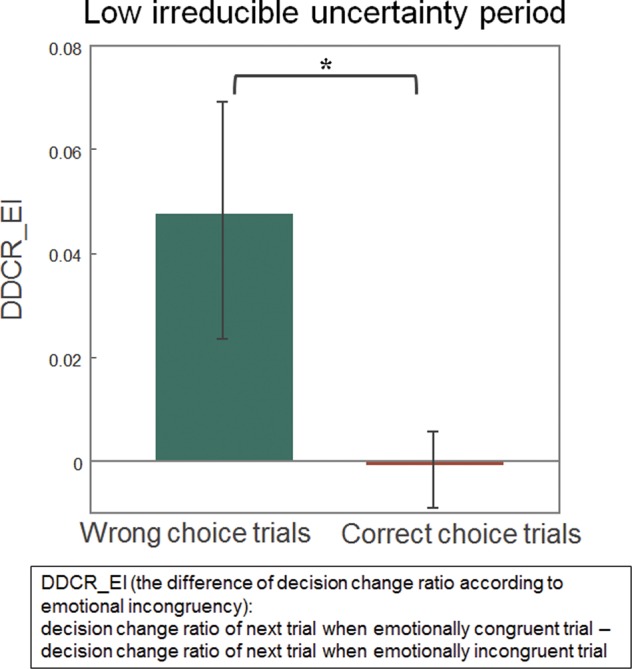
The difference in the decision change ratio according to emotional incongruency under the low irreducible uncertainty condition. To clarify the interaction effect between emotionally congruent feedback and the correctness of the choice on the decision change ratio, we compared the difference in the decision change ratio according to emotional incongruency during the incorrect choice trials and correct choice trials using a one-sample *t*-test. The effect of social noise on decision change was present under the low irreducible uncertainty condition (*t*-value (33) = 2.221, *p*-value = 0.033), *p-value < 0.05.

If social noise has a large effect, *DC_WC* − *DC_WI*, which is the decision change ratio difference after making an incorrect choice, would be large because the decision change was reduced by the emotionally incongruent feedback, while *DC_CC* − *DC_CI* would be large in the negative direction because emotionally incongruent feedback reduces the likelihood of making a choice consistent with that made in the previous trial. Therefore, SN_d would be large if the effect of social noise is large. Notably, we expected that the effect of social noise could be blurred by the effect of another type of uncertainty, such as irreducible uncertainty and volatility. In particular, our design included blocks with varying degrees of the association probability between the cue and outcome. In some blocks, this association probability was close to 50% to represent a situation with high irreducible uncertainty in which decision-making would be difficult. However, in the other blocks, the probability was close to 0% or 100% to represent situations with low irreducible uncertainty in which decision-making is easy. Considering these differences in uncertainty among the blocks of trials, we divided the trials into blocks with low irreducible uncertainty and high irreducible uncertainty. In particular, we divided all 300 trials into 150 trials with a probability assignment close to 50% (high irreducible uncertainty) and 150 trials with a probability assignment close to 0% or 100% (low irreducible uncertainty). Notably, in this analysis, we did not use the trial-by-trial uncertainty estimates derived from the computational modelling results. We performed two one-sample *t*-tests of SN_d separately according to the level of uncertainty. The results of the *t*-test showed that the effect of social noise was only present under the low irreducible uncertainty condition (*t*-value (33) = 2.221, *p*-value = 0.033; see Fig. 3) but not under the high irreducible uncertainty condition (*t*-value (33) = −0.422, *p*-value = 0.676).

### Computational model of behaviour under social noise: HGF-S model

The above results suggested that introducing social noise could influence the subjects’ decisions. We hypothesized that the influence of social noise on decision-making would be mediated by an altered generative model updating in the subjects. To test this hypothesis, we explicitly modelled the effect of social noise as perceptual uncertainty regarding sensory input using a modified version of the HGF, which we named HGF-S. Briefly, the HGF model assumes that an agent has a hierarchical model that generates sensory input and is updated hierarchically based on precision-weighted prediction error from a lower level of the hierarchy. In this HGF-S model, emotionally incongruent feedback, but not emotionally congruent feedback, induces perceptual uncertainty (social noise) regarding the sensory input (i.e., the valence of the feedback – correct or incorrect). Then, this perceptual uncertainty induces less updating of the belief at the first level of the hierarchy because of its unreliability compared to a situation with no perceptual uncertainty (emotionally congruent feedback), and the prediction error at this level decreases (for details, see Methods section). Importantly, because the updating of a belief is proportional to the prediction error occurring at the lower level of the hierarchy, the updating of the second level is influenced by the decreased prediction error at the first level, and the updating of the third level is also influenced similarly. Therefore, the effect of perceptual uncertainty propagates through the updating of all levels of the generative model, suggesting that social noise influences learning related to the belief at each level, including the volatility of the environment. Furthermore, the subsequent decision made using the prediction from this updated generative model could also be influenced. In the HGF-S model, the degree to which emotionally incongruent feedback induces perceptual uncertainty (social noise) is represented by the parameter *τ*.

### Model comparisons at the group level

To determine whether the HGF-S model explains the subjects’ behaviour better than other models that do not consider the effect of social noise, we compared four models (Sutton K1 [SK-1]; Rescorla-Wagner [RW]; HGF original; and HGF-S) using random-effects Bayesian model selection^4^. The results of the random-effects Bayesian model selection (HGF-S: exceedance probability: 0.993, protected exceedance probability: 0.993) showed that HGF-S had the best fit for PAL-S (see Supplementary Fig. S1).

### Relationship between social noise parameter τ and the subsequent decision change

We hypothesized that individuals with a high *τ* would be largely influenced by emotionally incongruent feedback in updating the generative model and, thus, that their subsequent decision would be significantly influenced. To test this hypothesis, we performed a correlation analysis between *τ* and SN_d under the low irreducible uncertainty condition. The results showed that SN_d and *τ* were significantly positively correlated (Spearman’s ρ = 0.406, p = 0.018) under the low irreducible uncertainty condition, suggesting that if emotionally incongruent feedback induces large social noise (large *τ*), the subsequent decision changes are also more likely to be influenced, which is consistent with our hypothesis.

### Simulation of behaviour using the HGF-S model

**Figure 4:**
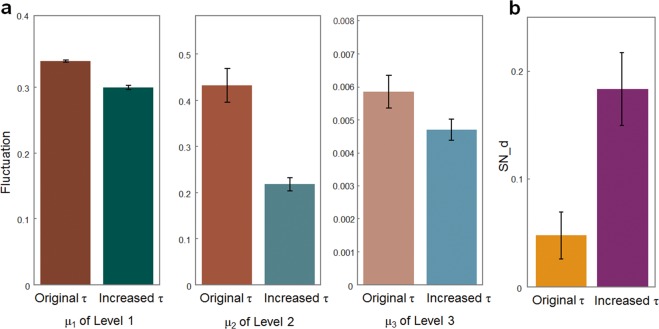
The changes between the original *τ* and the simulation with increased *τ*. (**a**) We artificially increased the subjects’ *τ* and analysed the difference in the belief fluctuation between the simulated responses and the original responses. At level 1, level 2, and level 3, as *τ* increased, the belief fluctuation in the simulation decreased more than the original response. **(b)** Under the low irreducible uncertainty condition, as *τ* increased, the SN_d in the simulation increased more than the SN_d of the original response. The error bars represent the standard error of the mean (SEM) across the subjects. SN_d (effect of Social Noise on Decision change): SN_d were quantified for each subject as an interaction effect between emotionally congruent feedback and the correctness of the choice on the decision change ratio, which was formulated as follows; *SN_d* = (*DC_WC* − *DC_WI)* − *(DC_CC* − *DC_CI), DC_WC:* Decision Change ratio after Wrong choice with emotionally Congruent feedback*, DC_WI:* Decision Change ratio after Wrong choice with emotionally Incongruent feedback*, DC_CC:* Decision Change ratio after Correct choice with emotionally Congruent feedback*, DC_CI:* Decision Change ratio after Correct choice with emotionally Incongruent feedback.

To further determine whether the HGF-S model explains the subjects’ behaviour under social noise, we performed the simulation 100 times using estimated parameters of each subject and tested whether the simulated response could recover, and the results of our behavioural analysis showed the effect of social noise on the decision change ratio (SN_d effect). The results showed that the simulated response closely recovered the behaviour pattern, illustrating the effect of social noise on the decision change ratio under the low irreducible uncertainty condition (t (3,399) = 20.18, p < 0.001). Furthermore, we artificially increased the subjects’ social noise parameter *τ* (by adding 3 to the original *τ*) and tested the difference in the behaviour patterns between the simulated responses and the subjects’ original responses (see representative subject in Supplementary Fig. S2). We hypothesized that perceptual noise decreases generative model updating and that the fluctuation in the subjects’ belief during the task would be decreased at every level of the belief. We quantified the fluctuation in the belief by the sum of the absolute difference in the belief between consecutive trials during the task at each level. For example, the fluctuation in the belief at level 1 is formulated as follows:

$$\sum _{k=1}^{N-1}\,|{\mu }_{1}^{(k+1)}-{\mu }_{1}^{(k)}|$$

where *N* is the number of total trials, and the *k* is the trial number. The same formula is applied to the fluctuation in the belief at levels 2 and 3. Consistent with our hypothesis, at level 1, level 2, and level 3, the fluctuation in the beliefs decreased in the simulation as *τ* increased compared to the original belief trajectory of the subjects (see Fig. 4a), suggesting that increasing social noise induced by emotionally incongruent feedback decreases learning at every level. Additionally, the trajectory of volatility at level 3 continued to decrease. Furthermore, we hypothesized that SN_d would also increase in the simulation as *τ* increased (see Fig. 4b). Under the low irreducible uncertainty condition, SN_d was even further increased (paired *t*-test comparing the difference in SN_d between the simulation with increased *τ* and the original subjects’ responses, *t*-value (33) = 3.09, *p*-value = 0.004), suggesting that as emotionally incongruent feedback induces more social noise, decisions are accordingly more strongly influenced by such feedback under low irreducible uncertainty. Additionally, to test the robustness of the HGF-S model, we performed a parameter recovery simulation and parameter correlation analysis (see Supplementary Methods).

### Emotional face perception-related MEG source is significantly related to the HGF-S parameters

The results of the computational modelling showed that the same emotionally incongruent feedback induced different amounts of social noise, which was represented by the model parameter *τ* of each subject. We suspected that these differences could be related to individual differences in the size of the neural response under the happy and sad face conditions. More specifically, we hypothesized that if neural processing differences within some regions, time points, and frequency bands are related to the amount of social noise induced by emotionally incongruent feedback, these cluster should satisfy the following conditions. As necessary conditions, (1) the ERD induced by the happy and sad faces should significantly differ, and (2) the difference in the ERD induced by the happy and sad faces in these clusters should be correlated with *τ*. To investigate our hypothesis and determine these clusters, we first determined the regions, time points, and frequency bands resulting in significant differences in ERD. Subsequently, we determined the regions, time points, and frequency bands resulting in differences in the ERD induced by the happy and sad faces that are significantly correlated with *τ*. Finally, we identified a cluster with significant differences in ERD and ERD differences significantly correlated with *τ*.

Using MEG source data, the brain regions, time points, and frequency bands with statistically significant areas in response to each emotional valence (happy or sad) were obtained through time-frequency decomposition and baseline normalization. The resulting values in the alpha frequency band were in the form of ERD, which is interpreted as the activation of an area^5^.

The ‘ERD difference’ clusters were calculated by a cluster-based permutation paired *t*-test across brain regions and time points and a Bonferroni correction across the frequency bands to correct for multiple comparisons (cluster-forming threshold = 0.01, cluster-level *p*-value < 0.025/6, Monte Carlo, randomization 10,000, n = 34). The following two statistically significant clusters were identified: alpha frequency bands in a part of the right hemisphere (Monte Carlo cluster *p*-value = 0.0008, cluster sum (*t*-value) = −4,934, see Supplementary Table S2) and alpha frequency bands in a part of the left hemisphere (Monte Carlo cluster *p*-value = 0.0014, cluster sum (*t*-value) = −3,357, see Supplementary Table S3).

**Figure 5:**
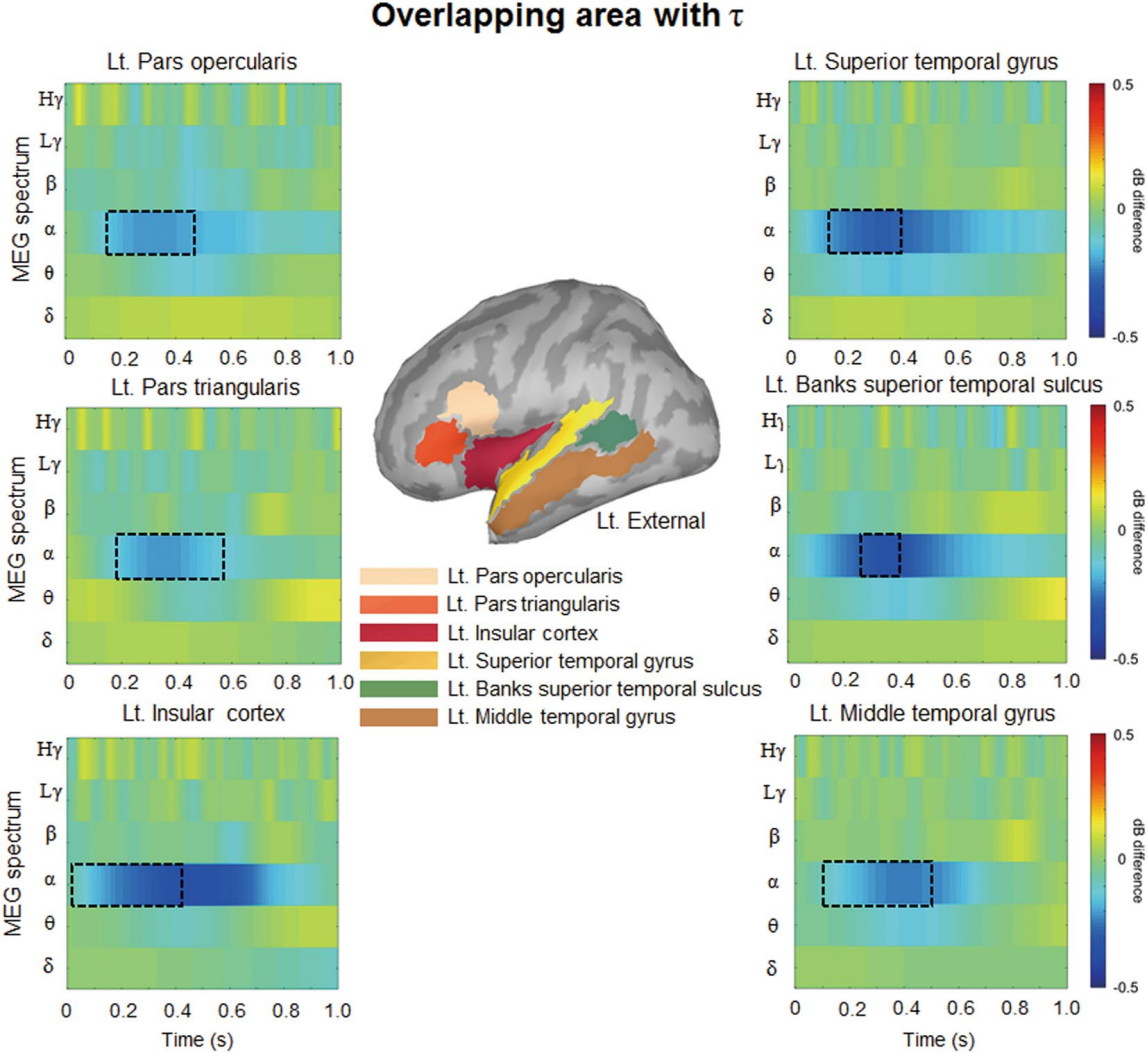
Time-frequency map of the ‘ERD difference’ and areas overlapping with τ (‘τ Cluster’). The overlapping areas appear in a part of the left hemisphere (the left transverse temporal cortex, left superior temporal gyrus, left insular cortex, left banks of the superior temporal sulcus, left pars opercularis, left pars triangularis, and left middle temporal gyrus), and the overlapping regions in frequency and time are represented by dotted rectangles. The upper middle shows the seven brain regions in the ‘τ Cluster’.

We calculated clusters with a significant correlation between ‘ERD difference’ and *τ* among ‘ERD difference’. The clusters were extracted by performing a cluster-based Pearson’s correlation analysis across brain regions and time points and correcting for multiple comparisons (cluster-forming threshold = 0.01, cluster-level *p*-value < 0.025, Monte Carlo, randomization 10,000, n = 34). The resulting clusters correlating with *τ* were obtained in the alpha frequency band (Monte Carlo cluster *p*-value = 0.0364, cluster sum (*t*-value) = 4,743, see Supplementary Table S4). However, these clusters failed to pass the Bonferroni correction (*p*-value < 0.025/6) reflecting the six frequency bands. Notably, the clusters showing significant correlations between *τ* and ‘ERD difference’ and the clusters with a significant ‘ERD difference’ overlapped in a specific time window, brain regions, and frequency band. Hereafter, we refer to these correlated areas as ‘*τ* Cluster’ (see Fig. 5, Supplementary Table S5, and Fig. S5), which include the left superior temporal gyrus (140~401 ms), left insular cortex (22~421 ms), left banks of the superior temporal gyrus (265~397 ms), left pars opercularis (151~487 ms), left pars triangularis (187~577 ms), and left middle temporal gyrus (104~507 ms). All significant regions were found in the alpha frequency band.

## Discussion

In a volatile environment, rapidly learning about a dynamically changing environment is essential for survival. However, if the feedback signal conveys unreliable information, learning about the environment might be disturbed. In this study, we investigated how feedback with social noise interferes with learning in a volatile environment by designing a PAL-S task reflecting this situation such that emotionally incongruent feedback induced social noise. In the behavioural analysis, using the quantity SN_d, we found that social noise affects subsequent decision-making, especially during low irreducible uncertainty trials. Moreover, the HGF-S model revealed that social noise induced by the emotionally incongruent feedback representing the perceptual uncertainty of the sensory input explained the subjects’ behaviour better than the other models that do not consider the effect of social noise. Furthermore, the correlation between *τ* and SN_d and the subsequent simulation results showed that the influence of social noise on the updating of the generative model also influences decision-making during subsequent trials. At the neural level, the model parameter *τ*, which reflects the degree to which emotionally incongruent feedback induces social noise in each individual, was significantly related to the individual differences in the ERD difference in several regions, including the right anterior insula and temporoparietal junction (TPJ) clusters, suggesting that the individual differences in processing emotional faces in these regions influence the amount of social noise induced by emotionally incongruent feedback.

These results further advance our understanding of the emotional interference effect on inference and learning. Previous studies using the emotional Stroop task^6^ or affective go/no-go task^7^ have shown how incidental emotion at the timing of a cue affects decision-making. However, in contrast to these studies investigating the emotional interference effect on decision-making at the time of a cue, our study investigated how feedback with emotional interference affects learning.

We clarified that the emotional congruency or incongruency of the feedback influenced the subjects’ subsequent choice during the next trial because the increased perceptual uncertainty induced less model updating by the sensory input. In particular, in the case of an incorrect choice, we found that emotionally incongruent feedback (a happy face) induces less decision change than emotionally congruent feedback because of the unreliable feedback signal (i.e., high perceptual uncertainty). In contrast, when the participants made a correct choice, we found that compared to emotionally congruent feedback, emotionally incongruent feedback (a sad face) increased decision change. This influence of social noise on decision change was quantified in each subject as an interaction effect between the feedback congruency and the correctness of the choice on the decision change ratio, i.e., the SN_d effect. Furthermore, the SN_d effect only exists in trials with low irreducible uncertainty and is absent in trials with high irreducible uncertainty. This result suggests that while social noise may have an effect on decision change, the SN_d effect might be quite small such that it could be masked by another type of uncertainty (irreducible uncertainty in this case).

By using a computational model of HGF-S, we found that social noise induced by emotionally incongruent feedback decreased the updating of the generative model, which also influenced the subsequent decision. In the HGF-S model, we initially expected that the decreased updating of the belief by social noise at the first level could be propagated to a higher level of the belief, including level 3, which encodes the volatility of the environment, through a decreased prediction error signal weighted by precision, thus suggesting that social noise induced by emotionally incongruent feedback could influence learning about the volatility of the environment. The results of the simulation performed in a previous study by Mathys *et al*. were consistent with this suggestion^8^. These authors showed that under a condition with high sensory precision (low sensory uncertainty), the change in belief was more ‘responsive’ to input, suggesting that compared to a condition with low sensory precision (high sensory uncertainty), under a condition with high sensory precision (high sensory uncertainty), beliefs are more likely to change towards the direction of sensory input. Moreover, under a low sensory precision condition, the authors showed that because most of the effect caused by changing sensory input is attributed to perceptual uncertainty rather than environmental volatility, the estimate of volatility at level 3 continues to decrease. Consistent with these previous simulation results and our suggestion above, by comparing the belief trajectory from the original *τ* of the subjects and the belief trajectory from the simulation with increased *τ*, we showed that the fluctuation in the belief at every level during the task decreased by increasing the social noise induced by the emotionally incongruent feedback (*τ*), suggesting that learning induced by feedback is decreased by social noise. Moreover, the volatility at level 3 continued to decrease, which is consistent with the results reported in a previous study^8^. The above results consistently suggest that in the HGF-S model, agents’ learning under volatility, i.e., the updating of the generative model by feedback, is decreased by social noise. Furthermore, according to the Bayesian model selection results, the HGF-S model explained the subjects’ behaviour better than the other models. Thus, social noise likely influenced the subjects’ learning in these ways. Furthermore, the analysis of the decision made during the subsequent trial strengthened our hypothesis. Because model updating influences the prediction of the generative model during the subsequent trial, we expected social noise during the previous trial to influence the decision during the subsequent trial, which was observed in our analysis showing the existence of the SN_d effect during the low irreducible uncertainty trials. Moreover, using a correlation analysis, we showed that SN_d reflecting the effect of social noise on the decision change pattern increased as *τ* increased, representing the increased social noise induced by emotionally incongruent feedback in an individual. Finally, the simulation results confirmed that increasing *τ* changed not only belief updating but also the subsequent decision. In summary, we show that social noise decreases learning about environmental states and the volatility of the environment by decreasing generative model updating at each level, which influences subsequent decision changes.

Considering that social noise induced by emotionally incongruent feedback represents uncertainty regarding the feedback, which is sensory input, such uncertainty might be understood as sensory uncertainty, such as visual uncertainty induced by random dot motion^9,10^. While many previous studies have shown how sensory uncertainty guides behaviour, such as sensorimotor control^11^, how such uncertainty affects learning is relatively under investigated. However, in this study, we showed that social noise could act as sensory uncertainty regarding feedback and influence learning, which is similar to the findings reported by Mathys *et al*.^8^. Furthermore, in the context of sensory uncertainty, this uncertainty could originate from both the uncertainty produced by the stimulus and the uncertainty produced by the sensory organs^10^. In the case of emotionally incongruent feedback, while the same stimuli were presented to all subjects as emotionally incongruent feedback, a different amount of social noise was produced, which was reflected in the variability of parameter *τ*. This finding suggests that the amount of social noise produced by the same emotionally incongruent feedback depends on an individual’s state or the traits of the brain. In particular, we suspect that the magnitude of the differential neural responses produced by the sad and happy faces could be related to individuals’ state or traits of the brain. To test this hypothesis, we first calculated the ‘ERD difference’ and statistically associated significant brain regions and time points. We also found regions in which the ERD differences were correlated with *τ*. As a result, we found overlapping brain regions and time points between a cluster showing a statistically significant ‘ERD difference’ and a cluster in which the ‘ERD difference’ and *τ* were statistically related. These regions, which we named the ‘*τ* cluster’, included the anterior insula and its surrounding regions and regions within the TPJ. Because the right anterior insula is known to encode the degree of emotion felt by a person^12,13^, our results might suggest that with larger ERD differences in the right anterior insula, the difference in the emotion felt in response to the sad and happy faces could be greater such that the sad face induced a more negative emotion, and the happy face induced a more positive emotion. Therefore, in this case, emotional face incongruency with feedback could induce a stronger emotional interference effect. Furthermore, the TPJ is known as a key node in the theory of mind network and social brain^12,14–16^. Therefore, the difference in the degree of emotion felt in response to others’ emotional faces might also induce social noise similar to that observed in the anterior insula. However, the direct testing of this hypothesis is impossible in our study because we did not acquire MEG recordings during the PAL-S task. Therefore, further experiments investigating the neural correlates of social noise during PAL-S should be performed to clarify this hypothesis.

Some limitations of this study are noteworthy. First, we did not measure the neural responses while the subjects performed the PAL-S. If the corresponding neural responses were measured during the PAL-S, we could have obtained various types of information regarding social noise in a volatile environment. However, measuring the emotional response felt by an individual performing PAL-S and confirming that the brain response is purely directed to the emotional faces is challenging and could represent a confounding factor. We believe that this confounding effect was prevented by the separation of the PAL-S and MEG experiments. However, because the cross-regression of the parameters of the two independent experiments performed using the same individuals showed a decrease in the expected effect size, we provided correlation coefficients instead of performing a regression analysis. Second, the possible effect depending on whether the gender selected by the subjects matched the gender in the feedback of the emotional faces was not considered as a major factor related to behaviour in our study. The main effect of the gender mismatch under the low irreducible uncertainty conditions was not statistically significant according to the repeated measures analysis of variance (ANOVA) [2 (correctness) × 2 (gender mismatch)] (see Methods section). Therefore, the gender mismatch effect may be limited. Third, the time courses of the correctness-gender association and the button-gender association (left button/female, right button/male) were not counterbalanced among the subjects.

In summary, by using computational modelling, we found that when social noise is induced by emotionally incongruent feedback in a volatile environment, learning about the environmental states and volatility is disrupted by a decreased prediction error signal caused by social noise. Moreover, we found that the amount of social noise induced by the same emotionally incongruent feedback differs among individuals. This finding was reflected by model parameter τ. The results of the ERD analysis suggest that the amount of emotional processing difference might induce this differential effect of emotionally incongruent feedback on the induction of social noise.

Our results provide an advanced understanding of the effect of distracting social information on learning in a volatile environment. Furthermore, as mentioned above, social noise produced by emotionally incongruent feedback, as reflected by *τ* differed among individuals, and this difference was related to the different ERD produced by the happy and sad faces among the subjects. These individual differences could be related to the subjects’ current psychological states, including major depressive disorder^17^ or borderline personality disorder^18^, or their past experiences, such as a history of childhood maltreatment or physical abuse^19,20^. From the perspective of computational psychiatry, future studies investigating the relationship between psychiatric diseases or psychiatric history influencing the current status and the degree of social noise produced by distracting social information could be beneficial to enhancing our understanding of altered emotion processing in these cases.

## Methods

### Subjects

Forty healthy subjects were enrolled in our experiment. The subjects were interviewed by psychiatrists using the Diagnostic Interview for Genetic Studies. The subjects had no history of psychiatric or neurological illness and had normal or corrected vision. All subjects provided written informed consent. This research was approved by the relevant Korean Advanced Institute of Science and Technology Institutional Review Boards and was conducted in accordance with the Declaration of Helsinki. The subjects underwent MEG recordings, behaviour tests, and structural MRI scans. The MEG recording failed in five subjects as follows: the recording failed in four subjects due to noise from dental prostheses and one subject due to excessive head movements. One subject was excluded because the digitizer failed, causing the information regarding the subject’s head position to be lost. Finally, after the exclusion of these six subjects, data from 34 subjects (age [mean ± standard deviation]: 23.94 ± 3.37 years, male/female: 16/18) were analysed. The Korean-Wechsler Adult Intelligence Scale was used to measure the subjects’ IQ (mean ± standard deviation: 121.18 ± 10.45).

### Probabilistic associative learning with social noise

The subjects performed the PAL-S in a soundproof room. We adopted the auditory-visual probabilistic association task, which was successfully modelled in a previous study^3^. During the task, the degree of the association between the auditory (low or high tones) and visual (female or male faces) stimuli changed over time.

In the PAL-S, the subjects heard low or high tones and predicted whether a female (F) or a male (M) match the low or high tone sounds. The probability of a woman’s face appearing after a high sound, i.e., p(F|HT), was changed over time. The change in probability p(F|HT) over time in the PAL-S is shown in Fig. 1a. All 300 trials were divided into low irreducible uncertainty trials (150 trials) with a probability assignment close to 0 or 100% and high irreducible uncertainty trials (150 trials) with a probability assignment close to 50%. Thus, the subjects learned about the association between the sound tone and gender in a volatile environment and about how ‘volatile’ the environment is. The subjects decided whether to press either the left (female) or right (male) button based on the current sound cue and then received feedback. Before the task, the subjects were informed that the associations between the tone and gender were probabilistic and that the expression of the face presented during the feedback could be irrelevant to their task. Furthermore, the subjects were informed whether they predicted correctly or incorrectly by the presence or absence of an “X” mark on the face images, and if the “X” feedback did not match the emotional face or gender feedback, the subjects were informed to trust the “X” feedback. We previously defined social noise as perceptual uncertainty caused by emotionally incongruent feedback. The emotionally incongruent feedback trial (correct answer-sad face, incorrect answer-happy face) and emotionally congruent feedback trial (incorrect answer-sad face, correct answer-happy face) were distinguished based on the congruency between the right/wrong valence and happy/sad valence. Two types of facial emotions (happy and sad) were randomly presented during feedback regardless of the correctness of the answer. Therefore, the trials were separated into two groups, namely, emotionally congruent and incongruent feedback trials [emotionally congruent feedback trial: (51.0% ± 0.02), emotionally incongruent feedback trial: (49.0% ± 0.02)]. Sad and happy faces were chosen for the comparison because sad and happy emotions cause low arousal and are at both ends of emotion valence.

We intended for this planned emotionally incongruency to cause perceptual uncertainty. This increased perceptual uncertainty could reduce model updating by the sensory input and affect the subjects’ decision during the subsequent trial. The subjects were asked to outperform the group average score to obtain additional monetary compensation and score higher than the results of randomized selection on the behavioural task.

The possible effect of whether the gender selected by the subjects matched the gender used in the feedback on the emotional faces was not considered a major factor affecting behaviour in our study [gender-expression congruent and emotionally congruent trial (20.2% ± 0.03), gender-expression incongruent and emotionally congruent trial (30.8% ± 0.03), gender-expression congruent and emotionally incongruent trial (19.2% ± 0.03), gender-expression incongruent and emotionally incongruent trial (29.8% ± 0.03)]. To confirm the effect of whether the gender selected by the subjects matched the gender on the feedback, we performed a 2 (correctness) × 2 (gender mismatch) repeated-measures ANOVA of the low irreducible uncertainty condition to determine the difference in the decision change ratio between emotional congruency and incongruency as a dependent variable. Based on the results, the main effect of gender mismatch was not significant (*F* (1,33) = 3.065, *p*-value = 0.089).

Before performing the PAL-S, the subjects practiced with a similar PAL with no social noise. The experimental procedures consisting of sound cues (0.3 s), response (1.15 s), feedback (0.15 s), and display of a fixation cross for a variable duration (1.1~2.2 s, mean: 1.64 s) are shown in Fig. 1b. The subjects were instructed to respond as quickly as possible within the given time (1.15 s).

### Behavioural modelling

PAL is an example of an advanced learning process in which people learn about the connection among stochastic external stimuli. When external stimuli change stochastically, sophisticated tasks and analytical techniques are required to investigate the influence of emotional factors on associative learning between stimuli. This type of learning has been successfully explained using classical conditioning models, such as RW and SK-1, and Bayesian inference models, such as HGF. We adopted the three abovementioned models, i.e., RW, SK-1, and HGF, to explain individuals’ learning in PAL-S. All three models are implemented in the HGF module in version 5.1 of the TAPAS (TNU Algorithms for Psychiatry-Advancing Science) toolbox (https://www.tnu.ethz.ch/en/software/tapas.html). The RW model^21^ is known to update beliefs by prediction errors under a fixed learning rate, and the SK-1 model^22^ is characterized by a learning rate change according to recent prediction errors. The HGF-S (see Fig. 2) was fitted to each individual’s choices using variational Bayes using three free perceptual-model parameters, namely, *ω*_2_, *ω*_3_, and *τ*, and response model parameter *ξ*.

In the HGF model, an agent uses sensory information to update its existing generative model and applies that model to make new decisions and responses^8,23^. The model assumes that the hidden states of the environment evolve as a Gaussian random walk and that the step size is affected by the level above. The links between each level are formulated as follows:

1 $${\rm{p}}({x}_{1}^{(k)}|{x}_{2}^{(k)})={s{({x}_{2}^{(k)})}^{{x}_{1}^{(k)}}(1-s({x}_{2}^{(k)}))}^{1-{x}_{1}^{(k)}}={\rm{Bernoulli}}({x}_{1}^{(k)};s({x}_{2}^{(k)}))$$

2 $${\rm{p}}({x}_{2}^{(k)}|{x}_{3}^{(k)})={\rm{N}}({x}_{2}^{(k)};{x}_{2}^{(k-1)},\,\exp (\kappa {x}_{3}^{(k)}+{\omega }_{2}))$$

3 $${\rm{p}}({x}_{3}^{(k)})={\rm{N}}({x}_{3}^{(k)};{x}_{3}^{(k-1)},{\omega }_{3})$$

where *s* is the sigmoid function and is represented as follows:

$$s(x)=\frac{1}{1+\exp (x)}$$

${x}_{1}^{(k)}$ represents an environmental state that generates sensory input following a Bernoulli distribution, ${x}_{2}^{(k)}$ represents the tendency of ${x}_{1}^{(k)}$ towards 1, and ${x}_{3}^{(k)}$ represents the inferred log-volatility of the environment reflecting changes in the correct probability given a cue. The parameter *κ* describes the influence of the third level on the second level, and we fixed this parameter to 1. The parameter *ω*_2_ reflects the tonic component of environmental volatility, which was estimated as a free parameter, and *ω*_3_ reflects the variance in volatility at level 3, which is occasionally called ‘meta-volatility’ and was estimated as a free parameter.

Inverting this model given the sensory input using the variational Bayesian scheme results in a set of closed-form update equations that provide a trial-by-trial update of the state variables in a hierarchical fashion in which the belief at each level is updated by the precision-weighted prediction error, which is formulated as follows:

4 $${\mu }_{2}^{(k)}-{\mu }_{2}^{(k-1)}={\sigma }_{2}^{(k)}{\delta }_{1}^{(k)}$$

${\delta }_{1}^{(k)}$ represents the prediction error at trial *k*, and ${\sigma }_{2}^{(k)}$ is the variance at the second level.

5 $${\delta }_{1}^{(k)}={\mu }_{1}^{(k)}-{\hat{\mu }}_{1}^{(k)}$$

${\hat{\mu }}_{1}^{(k)}$ is the prediction of the correct choice and is derived from a sigmoidal transformation of the previous belief about the probability of a correct choice ${\mu }_{2}^{(k-1)}$ as follows:

6 $${\hat{\mu }}_{1}^{(k)}=s({\mu }_{2}^{(k-1)})$$

At level 3, the update is proportional to the precision-weighted prediction error.

7 $${\mu }_{3}^{(k)}-{\mu }_{3}^{(k-1)}\propto \,{\psi }_{3}^{(k)}{\delta }_{2}^{(k)}=\frac{{\hat{\pi }}_{2}^{(k)}}{{\pi }_{3}^{(k)}}{\delta }_{2}^{(k)}$$

where ${\psi }_{3}^{(k)}$ is the precision ratio, and ${\delta }_{2}^{(k)}$ is the volatility prediction error, which is formulated as follows:

8 $${\delta }_{2}^{(k)}=\frac{{\sigma }_{2}^{(k)}+{({\mu }_{2}^{(k)}-{\mu }_{2}^{(k-1)})}^{2}}{{\sigma }_{2}^{(k-1)}+\exp (\kappa {{\mu }_{3}}^{(k-1)}+\omega )}-1$$

If there are no perceptual uncertainties, categorical environmental states ${x}_{1}^{(k)}\in \{0,1\}$ cause the same sensory input *u*^(*k*)^ such that ${x}_{1}^{(k)}={u}^{(k)}$, and the updated belief at level 1 at trial *k* (${\mu }_{1}^{(k)}$) is the same as *u*^(*k*)^ because an agent fully believes the sensory input, and this model has been used in many previous studies^3,24^. Therefore, in our model using emotionally congruent feedback such that there are no perceptual uncertainties, we used the belief updating equation ${\mu }_{1}^{(k)}={u}^{(k)}$ at level 1.

However, if perceptual uncertainty, such as social noise, exists, an agent cannot fully rely on sensory input to update the belief. Therefore, in the HGF-S model, we modelled social noise as perceptual uncertainty such that an environmental state that generates sensory input (${x}_{1}^{(k)}$) could cause sensory input *u*^(*k*)^ following a Gaussian distribution centred at ${x}_{1}^{(k)}$ with constant variance exp(*τ*)/2 as follows:

9 $${\rm{p}}({u}^{(k)}|{x}_{1}^{(k)})=N{({u}^{(k)};1,\frac{\exp (\tau )}{2})}^{{x}_{1}^{(k)}}\ast N{({u}^{(k)};0,\frac{\exp (\tau )}{2})}^{1-{x}_{1}^{(k)}}$$

Therefore, a larger *τ* indicates larger perceptual uncertainty. By using the derivation from Mathys *et al*.^23^, we obtain the following belief updating equation at level 1:

10 $${\mu }_{1}^{(k)}=\frac{\exp (-\frac{{({u}^{(k)}-1)}^{2}}{\exp \,(\tau )})\,\ast \,{\hat{\mu }}_{1}^{(k)}}{\exp (-\frac{{({u}^{(k)}-1)}^{2}}{{\exp }\,(\tau )})\,\ast \,{\hat{\mu }}_{1}^{(k)}+\exp (-\frac{{({u}^{(k)}-0)}^{2}}{{\exp }\,(\tau )})\,\ast \,(1-{\hat{\mu }}_{1}^{(k)})}$$

In contrast to the case of belief updating at level 1 with no perceptual uncertainties, in this update equation, ${\mu }_{1}^{(k)}$ is not the same as sensory input *u*^(*k*)^; in contrast, if *u*^(*k*)^ is 1, ${\mu }_{1}^{(k)}$ is slightly smaller than 1, while if *u*^(*k*)^ is 0, ${\mu }_{1}^{(k)}$ is slightly larger than 0. Furthermore, because HGF models the hierarchical updating of a belief using the prediction error from a lower level, the difference in updating at level 1 subsequently affects updating at higher levels. More specifically, in Eq. (5), assuming that sensory input *u*^(*k*)^ is 1, because prediction error ${\delta }_{1}^{(k)}$ with no perceptual uncertainty ($1-\,{\hat{\mu }}_{1}^{(k)}$) is larger in the direction of the sensory input (which is 1) than the prediction error with perceptual uncertainty (${\delta }_{1}^{(k)}={\mu }_{1}^{(k)}-{\hat{\mu }}_{1}^{(k)},{\mu }_{1}^{(k)} < 1)$, the subsequent updating at level 2

11 $${\mu }_{2}^{(k)}={\mu }_{2}^{(k-1)}+{\sigma }_{2}^{(k)}{\delta }_{1}^{(k)}$$

is also decreased (in the direction of the current input) in the case of perceptual uncertainty and no perceptual uncertainty. Furthermore, this decreased updating at level 2 influences the updating at level 3, and this logic is applied to the case when *u*^(*k*)^ is 0. In summary, in the HGF-S model, compared to the condition with no social noise (emotionally congruent feedback), social noise induced by emotionally incongruent feedback at the first level provokes less updating of the belief towards the direction of the sensory input at all levels of the hierarchy, which could influence the decision at the subsequent decision because the hierarchical prediction at the next trial depends on the updating of the previous trial. The decreased updating by perceptual uncertainty is also tested in the simulation results. Notably, the update equation in the case of perceptual uncertainty was applied to the trials with emotionally incongruent feedback. Finally, the response model calculating the choice probability p(*y*^(*k*)^ = 1) of the subjects was modelled using a unit square sigmoid function as follows:

$${\rm{p}}({y}^{(k)}=1)=\frac{{({\hat{\mu }}_{1}^{(k)})}^{{\rm{\zeta }}}}{{({\hat{\mu }}_{1}^{(k)})}^{{\rm{\zeta }}}+{(1-{\hat{\mu }}_{1}^{(k)})}^{{\rm{\zeta }}}}$$

where ζ is the inverse decision noise parameter such that increasing ζ decreases the stochasticity of the responses.

### Model comparison

To confirm that the behavioural tasks used in the experiments are appropriate for analysis with HGF-S, we compared HGF-S with the original HGF model, the RW model, and the SK-1 model. An observation model of a unit-square sigmoid was used for each learning model. The model comparisons were performed using a random-effect analysis for random-effects Bayesian model selection implemented in the VBA toolbox (mbb-team.github.io/VB A-toolbox/). The random-effects Bayesian model selection quantifies the evidence of a difference in model frequencies, such as estimated model frequencies and exceedance probabilities^4^. This method also provides protected exceedance probabilities that correct exceedance probabilities for the possibility that the observed differences in the models of the subjects are due to chance^25^.

### Experimental procedure during MEG scan

Because it is challenging to obtain electrophysiological data related purely to emotional face processing in a complex learning task with volatility, we performed the MEG recording and emotional perception task independently in a passive viewing task. The stimuli consisted of 45 faces and 45 text-based emoticons. The faces (15 happy, 15 sad, and 15 neutral faces) from the Korean Facial Expressions of Emotion database^26^ were used. Some emotional faces were also used as social noise in PAL-S (four happy and four sad faces). The happy and sad faces trials were used to identify emotional face processing-related brain activity during this emotional face perception task. The stimuli were standardized in independent samples of healthy volunteers (details are provided in the Supplementary Methods). The stimuli were shown using a projector. A photodiode was used to confirm the triggering point in each trial. A stimulus was displayed on the screen for 500 ms, and the stimulus onset asynchrony was 1,500 ms. The subjects performed 4 runs, each consisting of 180 stimuli (30 happy faces, 30 sad faces, 30 neutral faces, 30 happy emoticons, 30 sad emoticons, and 30 neutral emoticons) and lasting 270 s. Each run was separated by 5-minute breaks. In addition, when a question mark was presented, the subjects were asked to discriminate the emotion (happy or sad) displayed immediately before pressing the buttons. This question mark was randomly presented every 9 to 15 stimuli. This request motivated the subjects to encode the emotions of the emotional stimuli and concentrate on the experiment.

### MEG data acquisition and processing

The MEG data were acquired using a whole-head 152-channel MEG system (152 axial first-order double-relaxation oscillation superconducting quantum interference device gradiometers) at a sampling rate of 1,024 Hz in a magnetically shielded room. The attached positioning coils on the scalp determined the relative positions of the MEG sensors and the head in each block. Fiducial marks and the positioning coils enabled the coordination of the MEG data and T1-weighted structural MRI within subjects. The difference in the head position among the blocks did not exceed 2 mm, and the goodness of fit exceeded 95%. The analysis of the MEG data was performed using Fieldtrip^27^ (http://www.fieldtriptoolbox.org/) and Brainstorm^28^ (http://neuroimage.usc.edu/brainstorm). Using the Fieldtrip toolbox, the raw MEG signals were epoched from −700 to +1,300 ms relative to the stimulus onset, and baseline correction (−300~0 ms), power-line (60 Hz) notch filtering, detrending, and bandpass filtering (1 Hz and 100 Hz) were performed. Trials containing non-cortical physiologic activity or non-physiologic artefacts were removed by visual inspection. An independent component analysis using the function ‘ft_componentanalysis’ with the runica algorithm was performed by two trained neurologists to remove eye movement and heartbeat artefacts. The preprocessed epoched trials were downsampled at 256 Hz and then imported into Brainstorm for source reconstruction. First, the cortical surfaces of all subjects were generated from T1-weighted images acquired using a Siemens 3T Verio scanner (Erlangen, Germany), and 15,000 dipoles were created on the surface model. The initial MRI/MEG registration was improved using digitized head points obtained from the MEG acquisition. A forward model was calculated using the realistic overlapping spheres model. The source atlas was applied to the FreeSurfer’s Desikan-Killiany Atlas^29^, and 68 cortex surfaces were parcellated. The cortical activity was calculated as a weighted minimum-norm estimation in all trials. To obtain the time-frequency map, we used the Morlet wavelet convolution for all trials. Frequency was defined as delta (2–4 Hz), theta (5–7 Hz), alpha (8–12 Hz), beta (15–29 Hz), low gamma (30–50 Hz), or high gamma (70–90 Hz). The time points were sampled at 3 ms increments, and the average value was calculated according to the band range. A time-frequency map of all trials was obtained, and averaging was performed within each subject under the sad, happy, and neutral face conditions. After averaging the trials, the time-frequency map was normalized for each frequency at −300 ms~0 ms, and the unit was a decibel (dB). The above baseline normalization was necessary to identify the ERD^30,31^ and event-related synchronization (ERS)^32^. Using these ERD and ERS methods, we obtained the activity arising from local interactions between main neurons and interneurons that regulate the frequency components of the ongoing MEG (ERD)^5^. Because ERD in the alpha spectrum indicates a gradual release of inhibition related to activation^33^, the individual difference in ERD in the alpha spectrum was related to the difference in the activation of the clusters. Because we were interested in the neurophysiologic degree of the difference in emotional valence, we used the absolute value of the differences in ERD for the contrast between the happy and sad face conditions.

### Correction for multiple comparisons in the correlation analyses between the MEG differences and HGF-S parameters

The differences in the brain regions, time points, and frequency bands between the sad and happy emotional conditions were tested using a cluster-based permutation paired *t*-test (cluster-forming threshold = 0.01, cluster-level *p*-value < 0.025/6, Monte Carlo, randomization 10,000, n = 34) with correction for multiple comparisons. Specifically, regarding the cluster-based permutation paired *t*-test, first, we performed paired *t*-tests at each time point between 0 and 1,000 ms all 68 brain regions. Second, we clustered the significant spatiotemporal points with uncorrected *p*-values below 0.01 according to the spatiotemporal distance. Third, we summed the *t*-values of each cluster. Fourth, we calculated the permutation distribution with a random switching condition within the subjects and performed a paired *t*-test between the permutated conditions to form clusters. We repeated this permutation 10,000 times. Finally, based on this permutation distribution, we calculated the corrected *p*-values of the original clusters. All statistical tests were two-sided, and Bonferroni correction for multiple comparison was performed across the six frequency bands (0.025/6) because the spectrum was averaged by the frequency bands. A Pearson’s correlation analysis between *τ* and the ‘ERD difference’ was also performed using the same method applied to correct for the multiple comparisons. All analyses were performed in R (version 3.5.0)^34^ and MATLAB (R2016b).

## Supplementary information

Supplementary information

Dataset 1

## Data Availability

The data from the current study are available from the corresponding author upon reasonable request.

## References

[1] Xu M, Xu G, Yang Y (2016). Neural Systems Underlying Emotional and Non-emotional Interference Processing: An ALE Meta-Analysis of Functional Neuroimaging Studies. Front Behav Neurosci.

[2] Behrens TE, Woolrich MW, Walton ME, Rushworth MF (2007). Learning the value of information in an uncertain world. Nat Neurosci.

[3] Iglesias S (2013). Hierarchical prediction errors in midbrain and basal forebrain during sensory learning. Neuron.

[4] Stephan KE, Penny WD, Daunizeau J, Moran RJ, Friston KJ (2009). Bayesian model selection for group studies. Neuroimage.

[5] Pfurtscheller G, Lopes da Silva FH (1999). Event-related EEG/MEG synchronization and desynchronization: basic principles. Clin Neurophysiol.

[6] Williams JM, Mathews A, MacLeod C (1996). The emotional Stroop task and psychopathology. Psychol Bull.

[7] Erickson K (2005). Mood-congruent bias in affective go/no-go performance of unmedicated patients with major depressive disorder. Am J Psychiatry.

[8] Mathys CD (2014). Uncertainty in perception and the Hierarchical Gaussian Filter. Front Hum Neurosci.

[9] Kiani R, Shadlen MN (2009). Representation of Confidence Associated with a Decision by Neurons in the Parietal Cortex. Science.

[10] Bach DR, Dolan RJ (2012). Knowing how much you don’t know: a neural organization of uncertainty estimates. Nature Reviews Neuroscience.

[11] Orban G, Wolpert DM (2011). Representations of uncertainty in sensorimotor control. Current Opinion in Neurobiology.

[12] Adolphs R (1999). Social cognition and the human brain. Trends in Cognitive Sciences.

[13] Adolphs R, Damasio H, Tranel D, Damasio AR (1996). Cortical systems for the recognition of emotion in facial expressions. J Neurosci.

[14] Puce A, Allison T, Bentin S, Gore JC, McCarthy G (1998). Temporal cortex activation in humans viewing eye and mouth movements. Journal of Neuroscience.

[15] Allison T, Puce A, McCarthy G (2000). Social perception from visual cues: role of the STS region. Trends in Cognitive Sciences.

[16] Blakemore SJ (2008). The social brain in adolescence. Nat Rev Neurosci.

[17] Dalili MN, Penton-Voak IS, Harmer CJ, Munafo MR (2015). Meta-analysis of emotion recognition deficits in major depressive disorder. Psychol Med.

[18] Fenske S (2015). Emotion recognition in borderline personality disorder: effects of emotional information on negative bias. Borderline Personal Disord Emot Dysregul.

[19] Young JC, Widom CS (2014). Long-term effects of child abuse and neglect on emotion processing in adulthood. Child Abuse Negl.

[20] Masten CL (2008). Recognition of facial emotions among maltreated children with high rates of post-traumatic stress disorder. Child Abuse Negl.

[21] Black, A. H. & Prokasy, W. F. Classical conditioning II: Current research and theory (1972).

[22] Sutton, R. S. Gain adaptation beats least squares. In: *Proceedings of the 7th Yale workshop on adaptive and learning systems* (ed^(eds) (1992).

[23] Mathys C, Daunizeau J, Friston KJ, Stephan KE (2011). A bayesian foundation for individual learning under uncertainty. Front Hum Neurosci.

[24] Diaconescu AO (2017). Hierarchical prediction errors in midbrain and septum during social learning. Soc Cogn Affect Neurosci.

[25] Rigoux L, Stephan KE, Friston KJ, Daunizeau J (2014). Bayesian model selection for group studies - revisited. Neuroimage.

[26] Park, J. Y. *et al*. Korean Facial Expressions of Emotion (KOFEE). (ed^(eds). Section of Affect & Neuroscience, Institute of Behavioral Science in Medicine, Yonsei University College of Medicine (2011).

[27] Oostenveld R, Fries P, Maris E, Schoffelen JM (2011). FieldTrip: Open source software for advanced analysis of MEG, EEG, and invasive electrophysiological data. Comput Intell Neurosci.

[28] Tadel F, Baillet S, Mosher JC, Pantazis D, Leahy RM (2011). Brainstorm: a user-friendly application for MEG/EEG analysis. Comput Intell Neurosci.

[29] Desikan RS (2006). An automated labeling system for subdividing the human cerebral cortex on MRI scans into gyral based regions of interest. Neuroimage.

[30] Pfurtscheller G (1977). Graphical display and statistical evaluation of event-related desynchronization (ERD). Electroencephalogr Clin Neurophysiol.

[31] Pfurtscheller G, Aranibar A (1977). Event-related cortical desynchronization detected by power measurements of scalp EEG. Electroencephalogr Clin Neurophysiol.

[32] Pfurtscheller G (1992). Event-related synchronization (ERS): an electrophysiological correlate of cortical areas at rest. Electroencephalogr Clin Neurophysiol.

[33] Klimesch W, Sauseng P, Hanslmayr S (2007). EEG alpha oscillations: the inhibition-timing hypothesis. Brain Res Rev.

[34] R Core Team. R: A language and environment for statistical computing. (ed^(eds). R Foundation for Statistical Computing (2017).

