# Supplementary material for: Social noise interferes with learning in a volatile environment
Source: Sci Rep. 2019 May 20;9:7574. doi: 10.1038/s41598-019-44101-w (PMC6527564; doi:10.1038/s41598-019-44101-w)
Supplement: Supplementary file 1 — Supplementary information [file 41598_2019_44101_MOESM1_ESM.docx]

**Supplementary information**

Social noise interferes with learning in a volatile environment

Dong Woo Shin^1#^, Jaejoong Kim^1#^, Bumseok Jeong^1,2,4*^, Ko Woon Kim^1,3^, Geumsook Shim^4^, Taekeun Yoon^1^

## **Supplementary methods**

### **Standardization of the emotional stimuli**

Fifteen sad, fifteen happy, and fifteen neutral faces from the Korean Facial Expressions of Emotion database were standardized in an independent sample of 47 healthy volunteers (mean age = 28.43 ± 4.31 years, male/female: 26/21). The sad, happy and neutral face images were transformed to greyscale and masked by an oval form. The subjects in the independent samples were asked to rate the emotions they felt towards the picture stimuli on an 11-point Likert scale (-5 to +5). The emotion scores and their absolute values were calculated to confirm that there was no difference in the emotional valence magnitude between the happy and sad faces. The repeated-measures analysis of variance (RANOVA) of the 3 emotional valences (happy, sad, and neutral) showed a significant main effect of emotional valence (F(1.894, 87.131) = 123.939, *p*-value < 0.001, Greenhouse-Geisser corrected, n = 47). According to the post hoc *t*-tests, the emotional faces (happy and sad) showed significantly higher magnitudes of emotional valence than the neutral faces (*p*-value < 0.001). However, there was no significant difference in magnitude between the happy and sad faces (*p*-value = 0.172).

MEG recording was conducted during an emotion recognition task consisting of both passive viewing of faces and text-based emoticon stimuli and intermittent one-back questions regarding the type of emotion presented by the previous stimulus. In the current analysis, 45 face images (15 happy, 15 sad, and 15 neutral faces), but no emoticons, were used for the MEG recordings. Of the 30 happy and sad faces, 8 emotional faces consisting of 4 male and 4 female faces with happy or sad emotions were used as social noise in an independent behavioural task, i.e., PAL-S. Of the 30 happy and sad faces, 8 emotional faces with a happy or sad emotion were used as social noise in PAL-S. Of these 8 face images, the averaged emotion scores of the happy (1.97 ± 0.83) and sad (2.22 ± 0.89) faces were within one standard deviation of the means of the happy (2.17 ± 0.67) and sad (1.98 ± 0.82) faces among the 30 emotional face images. Among the 8 emotional face images, the main effect of emotion according to the RANOVA (F(1.962, 90.256 = 117.791), *p*-value < 0.001, Greenhouse-Geisser corrected, n = 47) was significant, and the post hoc *t*-tests comparing the happy and sad faced to the neutral faces were also significant (*p*-value < 0.001). There was no significant difference between the happy and sad faces (*p* -value = 0.080) in the magnitude of their emotional valence.

### **Parameter correlation**

A correlation analysis of the estimated parameters was performed to examine the model’s robustness. The results revealed a correlation between $\omega_{2}$ and $\zeta$ (Spearman’s $\rho$ = -0.36, p = 0.039). However, no parameters were correlated with $\tau$, which is our parameter of interest (all *p*-value > 0.3, see Supplementary Fig. S3 online).

### **Parameter recovery simulation**

To determine whether the parameters were estimated reliably, a parameter recovery simulation was performed by simulating the responses 100 times using estimated parameters of each subject and subsequently re-estimating the parameters based on these simulated responses. We obtained 100 correlation coefficients between the original parameters and each recovered parameter from 100 simulations. Then, we evaluated the means and standard deviations of the 100 correlation coefficients. The original model parameters were correlated with the recovered parameters (ω_2: Pearson’s r = 0.985, standard deviation: 0.005, p-value < 0.001, ω_3: Pearson’s r = 0.190, standard deviation: 0.244, p-value= 0.058, τ: Pearson’s r = 0.475, standard deviation: 0.239, p-value < 0.001, ζ: Pearson’s r = 0.933, standard deviation: 0.032, p-value < 0.001); see Supplementary Fig. S4 online), indicating that the parameters were reliably estimated.

## **Supplementary figures**


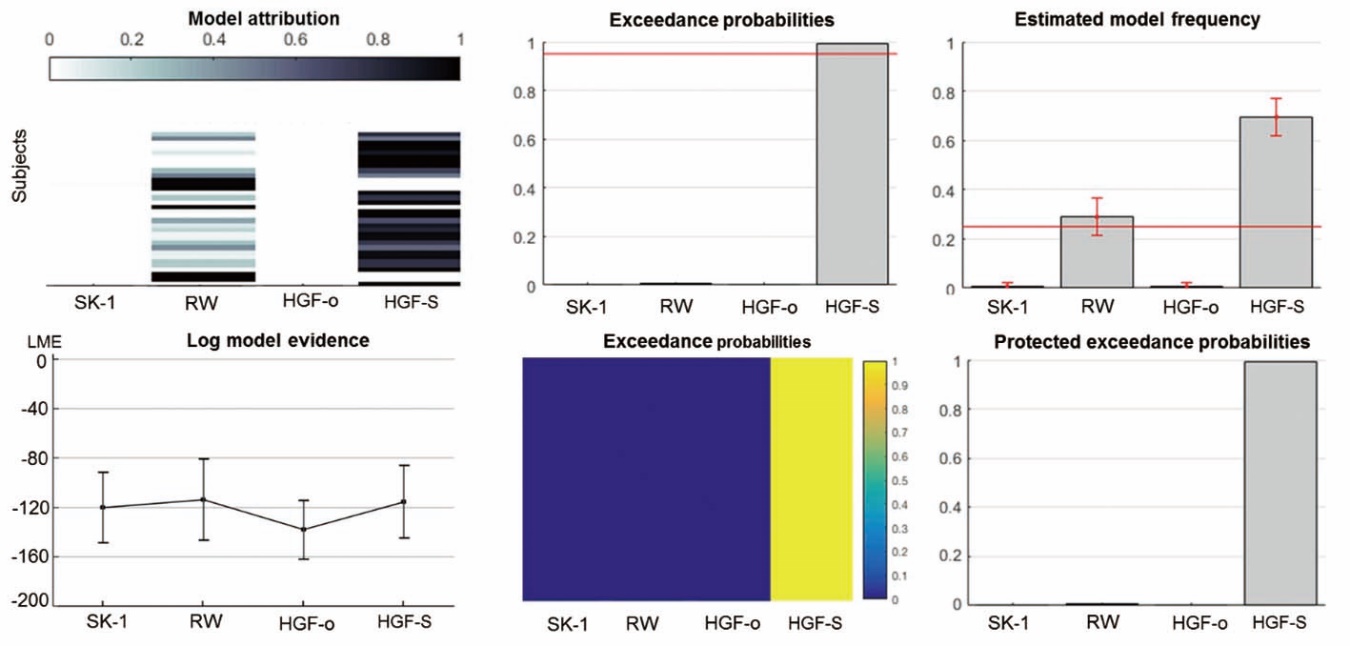


### **Supplementary Figure S1.** The results of the Random-Effects Model Comparison. We compared four models (SK-1, RW, HGF-o, and HGF-S) in a Random-Effects Model Comparison. The Random-Effects Model Comparison revealed an exceedance probability and protected exceedance probability of 0.993, suggesting that the HGF-S model was the best model for PAL-S. HGF: hierarchical Gaussian filter; RW: Rescorla-Wagner; SK-1: Sutton K1; HGF-o: HGF original; HGF-S: three-level hierarchical Gaussian filter with social noise.


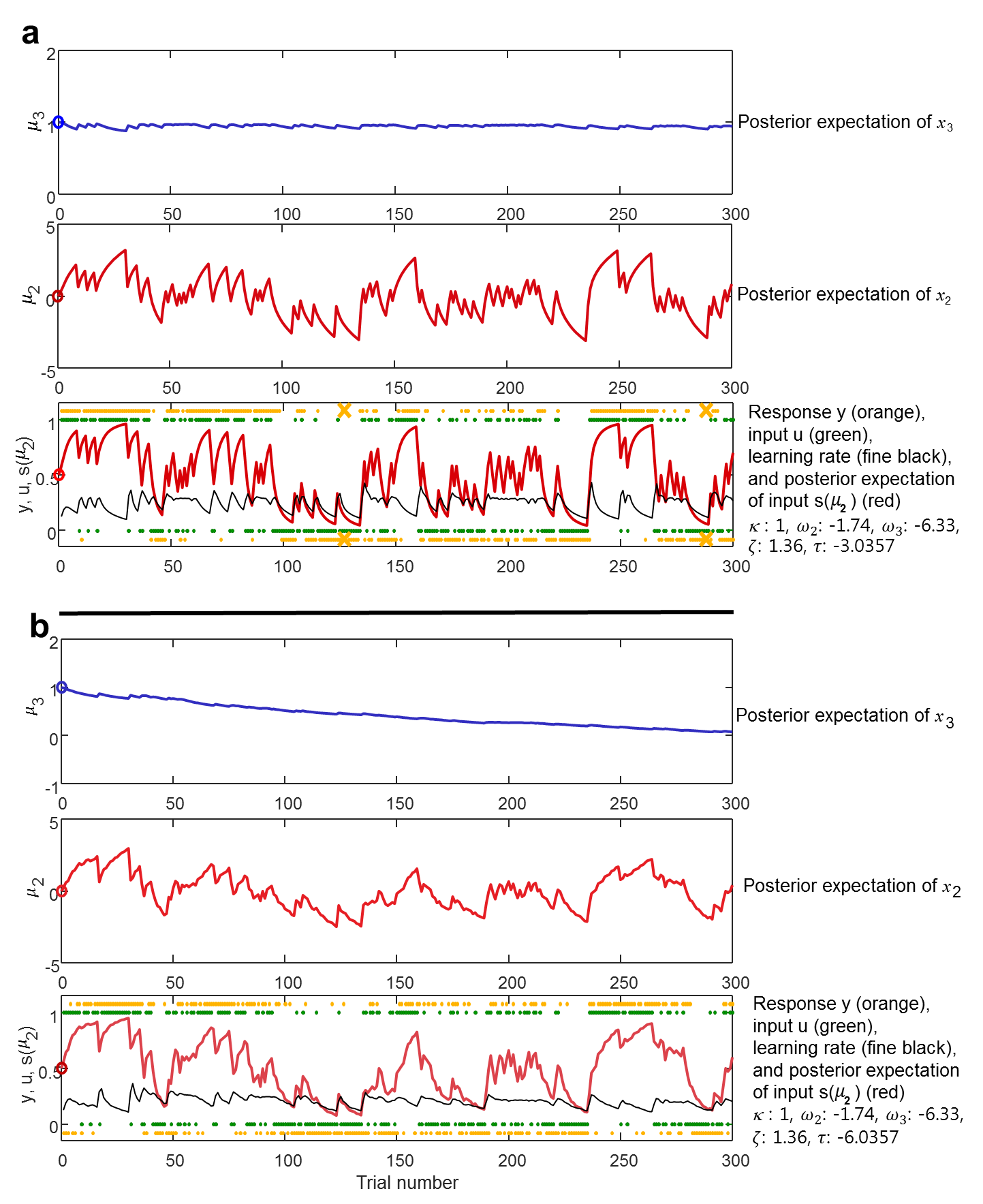


### Supplementary Figure S2. **The consequences of social noise in a representative subject.** **(a)** This figure shows the trajectories of the original $\tau$. **(b)** This figure shows the trajectories of the simulation results after adding 3 to the original $\tau$ (high $\tau$). During input prediction at level 1 and the belief tendency at level 2, the value fluctuations at a high $\tau$ were less than those at the original $\tau$. Moreover, at the belief volatility of level 3, we find a difference between the original $\tau$ and the simulated high $\tau$ condition.


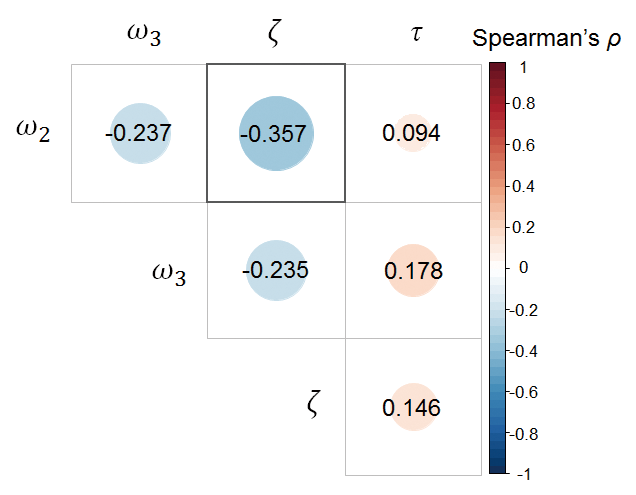


### **Supplementary Figure S3.** Spearman’s correlation analysis between the estimated parameters of the HGF-S model. Positive correlations are displayed in brown, and negative correlations are displayed in dark blue. The intensity of the colour and the size of the circle are proportional to the correlation coefficients, and these correlation coefficients are shown in the circles. On the right side, the matching of the colour and correlation coefficient is displayed. The results showed that $\omega_{2}$ and$\zeta$ are correlated (Spearman’s *ρ* = -0.357, p = 0.039). However, no parameters are correlated with $\tau$. Correlations with *p*-value < 0.05 are displayed in the bold grey box.

**
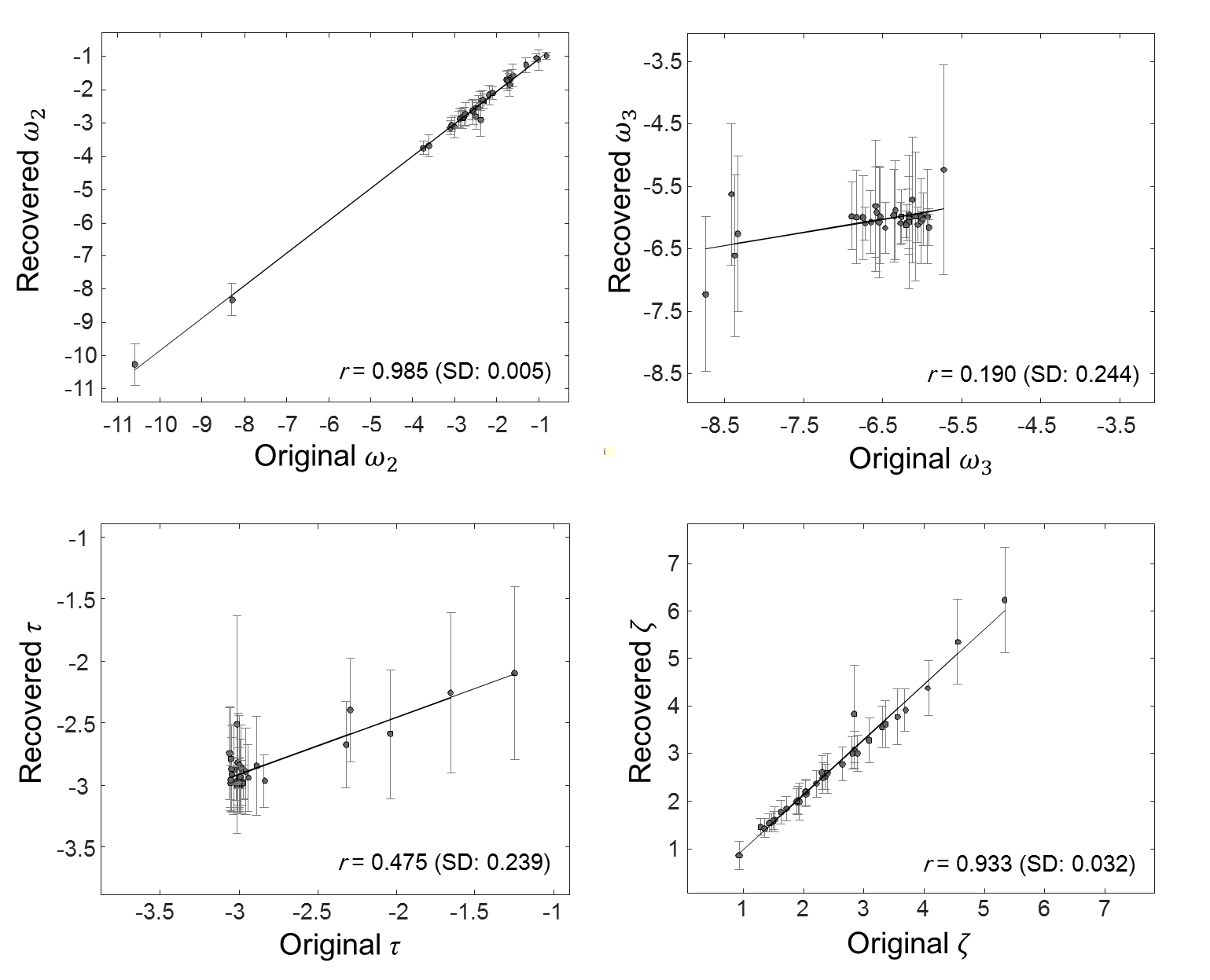
**

### **Supplementary Figure S4. Parameter recovery.** The original parameter is shown on the x-axis, and the recovered parameter is shown on the y-axis. The grey dot represents the point corresponding to the original parameter and the mean of the recovered parameter obtained from 100 simulations. We determined the error bars based on 34 subjects with standard deviations produced by 100 simulations. The r values shown in the figure represent the mean of 100 correlation coefficients (Pearson’s correlations) between the original parameter and the parameter obtained from each of the 100 simulations. The original model parameters were significantly correlated with the recovered parameters, indicating that the parameters were estimated reliably. SD: standard deviation.


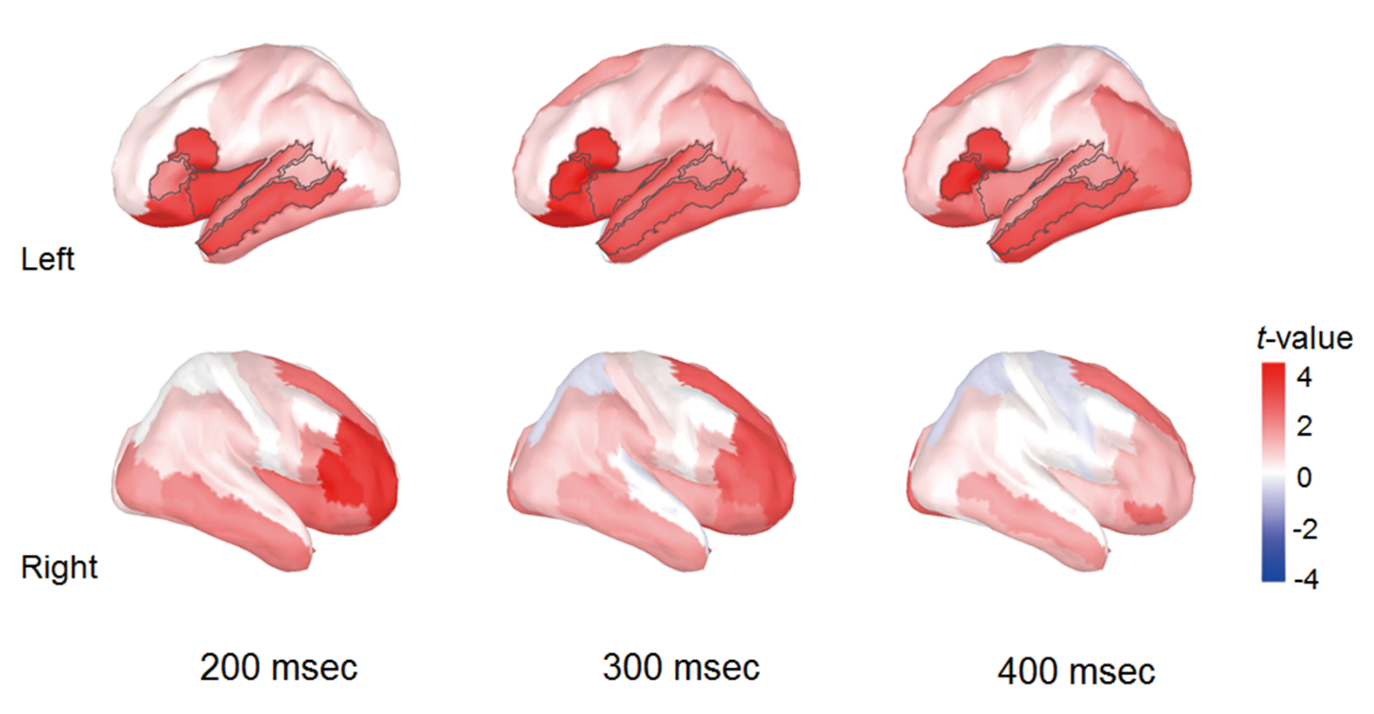


### **Supplementary Figure S5.** Statistical map of the cluster-based Pearson’s correlation between the ‘ERD difference’ and $\tau$ in alpha frequency at the source level. To solve the multiple comparison problem and identify statistically significant areas showing a correlation between $\tau$ and the ‘ERD difference’, including statistically significant and insignificant areas, we performed a cluster-based Pearson’s correlation analysis. Clusters with significant correlations between $\tau$ and the ‘ERD difference’ and clusters with a significant ‘ERD difference’ overlapping in a specific time window, brain regions, and frequency band (‘$\tau$ cluster’) are shown. The ‘$\tau$ clusters’ are represented by a dark grey line at 200, 300, and 400 ms.

## **Supplementary Tables**

### **Supplementary Table S1.** Descriptive statistics of the behaviour and HGF-S model parameters in PAL-S

SD: standard deviation

### **Supplementary Table S2.** Right cluster with a statistically significant ‘ERD difference’ in the alpha frequency band.

The cluster-forming alpha was 0.01, the cluster probability was 0.0008, and the cluster sum (*t*) was -4934. *: The cluster was retained after lowering the cluster-forming alpha to 0.001.

### **Supplementary Table S3.** Left cluster with a statistically significant ‘ERD difference’ in the alpha frequency band.

The cluster-forming alpha was 0.01, the cluster probability was 0.0014, and the cluster sum (*t*-value) was -3,357. *: The cluster was retained after lowering the cluster-forming alpha to 0.001.

### **Supplementary Table S4.** Cluster with a correlation between $\tau$ in PAL-S and the ‘ERD difference’, including statistically significant and insignificant areas, in the alpha frequency band

The cluster-forming alpha was 0.01, the cluster probability was 0.0364, and the cluster sum (*t*-value) was -4,743.

### **Supplementary Table S5.** Overlapping cluster (‘$\tau$ Cluster’) in the alpha frequency band between the statistically significant ‘ERD difference’ (Peak *t*(1)) and areas showing a correlation between $\tau$ in PAL-S and the ‘ERD difference’ (Peak *t* (2)).

*: The cluster was retained after lowering the cluster-forming alpha to 0.001 in ‘ERD difference’.

### **Supplementary Table S6.** Mean and variance of Gaussian priors used in the HGF parameter estimation.
